# Complete Genome Sequence of Enterotoxigenic Escherichia coli N4-Like Podophage Pollock

**DOI:** 10.1128/genomeA.01431-14

**Published:** 2015-01-29

**Authors:** Roosheel S. Patel, Lauren E. Lessor, Adriana C. Hernandez, Gabriel F. Kuty Everett

**Affiliations:** Center for Phage Technology, Texas A&M University, College Station, Texas, USA

## Abstract

Enterotoxigenic Escherichia coli is a multidrug-resistant bacterium that is well known for its ability to cause diarrhea in humans. Bacteriophages may be used to treat clinical cases involving bacterial dysentery. Here, we present the complete genome sequence of an enterotoxigenic E. coli phage, Pollock, an N4-like podophage.

## GENOME ANNOUNCEMENT

Enterotoxigenic Escherichia coli (ETEC) is a pathogenic type of E. coli associated with food/water contamination-related traveler’s diarrhea (1). With the rise of bacteria that are resistant to antibiotics, the need for alternative treatments is growing (2). One such alternative is the therapeutic use of virulent bacteriophages. Pollock is a newly isolated virulent N4-like podophage that has the potential to be used as a treatment against ETEC.

Bacteriophage Pollock was isolated from a sewage sample collected in College Station, TX. The phage DNA was sequenced using 454 pyrosequencing at the Emory GRA Genome Center (Emory University, GA). The trimmed FLX Titanium reads were assembled to a single contig at 157.7-fold coverage using the Newbler assembler, version 2.5.3 (454 Life Sciences), at the default settings. The contig was confirmed to be complete by PCR using primers that face the upstream and downstream ends of the phage DNA. The products from the PCR amplification of the junctions of concatemeric molecules were sequenced by Sanger sequencing (Eton Bioscience, San Diego, CA). The genes were predicted using GeneMarkS (3) and corrected using the software tools available on the Center for Phage Technology (CPT) Galaxy instance (https://cpt.tamu.edu/galaxy-public/). Morphology was determined using transmission electron microscopy performed at the Texas A&M University Microscopy and Imaging Center.

Pollock has a unit genome of 67,755 bp, including 84 predicted coding sequences, a G+C content of 36%, and a coding density of 93%. Four tRNA-coding sequences were also found. An analysis of the raw sequencing reads using PAUSE (https://cpt.tamu.edu/computer-resources/pause/) shows that Pollock has a 610-bp terminal repeat. Pollock is syntenic with E. coli podophage N4 (accession no. NC_008720) and has 49.9% identity to N4 via Emboss Stretcher analysis (4, 5).

Pollock encodes N4 homologs involved in DNA replication, transcription, DNA packaging, and morphogenesis. The defining characteristics of N4 are its large virion RNA polymerase (vRNAP), RNA polymerase II (RNAP II), and a single-stranded DNA binding protein (SSB) (6, 7). Pollock encodes the homologs of these proteins, suggesting that it accomplishes DNA injection and early gene expression by a similar mechanism. Unlike N4, however, the DNA polymerase of Pollock contains an intron-encoded HNH endonuclease (IPR003615) interrupting the coding sequence. The lysis cassette of Pollock is slightly different from that of N4, in that instead of a signal-anchor-release (SAR) endolysin, as encoded by N4 (*N*-acetylmuramidase), Pollock encodes a cytoplasmic endolysin (glycoside hydrolase family 24) (8, 9). Other lysis genes identified in Pollock include a holin/antiholin pair and an embedded inner/outer spanin pair (10).

Curiously, Pollock also encodes a homolog of the bacteriophage P1 protein, TciA (tellurite or colicin resistance or inhibition of cell division) (11). P1 *tciA* is homologous to *terB*, a gene from the *ter* gene cluster on the incompatibility subgroup IncHI2 R478 multiresistance plasmid. The *ter* genes are thought to confer E. coli resistance to various stresses, including channel-forming colicins and the propagation of certain phages (12, 13). How this protein plays a role in a presumably lytic N4-like phage is unknown.

### Nucleotide sequence accession number.

The genome sequence of phage Pollock was deposited in GenBank under the accession no. KM236242.
